# The Effect of HBV Genotype C on the Development of HCC Differs Between Wild-Type Viruses and Those With BCP Double Mutations (T^1762^A^1764^)

**DOI:** 10.5812/hepatmon.16214

**Published:** 2014-02-17

**Authors:** Qin-Yan Chen, Tim J Harrison, Caroline A Sabin, Guo-Jian Li, Gao-Ming Huang, Jin-Ye Yang, Xue-Yan Wang, Hai Li, Mo-Han Liu, Zhong-Liao Fang

**Affiliations:** 1 Guangxi Zhuang Autonomous Region Center for Disease Prevention and Control, Nanning, China; 2 Division of Medicine, Faculty of Medical Sciences, University College London, London, UK; 3 Research Department of Infection and Population Health, UCL Medical School, London, UK; 4 Department of Public Health of Guangxi Zhuang Autonomous Region, Nanning, China; 5 School of Public Health, Guangxi Medical University, Nanning, China; 6 School of Preclinical Medicine, Guangxi Medical University, Nanning, China

**Keywords:** Hepatitis B Virus, Genotypes, Basal Core Promoter Double Mutations, Hepatocellular Carcinoma

## Abstract

**Background:**

Association of hepatitis B virus (HBV) genotype C with hepatocellular carcinoma (HCC) development remains controversial. HBV basal core promoter (BCP) double mutations (T^1762^A^1764^) are very strong confounding factors of genotypes B and C in HCC development.

**Objectives:**

To investigate the association of HBV genotype C with HCC development after controlling for BCP double mutations.

**Materials and methods:**

Four hundred and two serum samples from patients with HCC, liver cirrhosis (LC) and chronic hepatitis (CH) and also from asymptomatic HBsAg carriers were analyzed.

**Results:**

Genotypes B (31.1%), C (62.8%), and I (6.1%) were detected. With the severity of liver disease the prevalence of genotype B decreased, but genotype C increased. No trend was found for genotype I. The prevalence of BCP double mutations in genotypes C and I viruses was significantly higher than genotype B. BCP double mutations are risk factors for CH, LC and HCC. Genotype C was not identified as a particular risk factor for HCC prior to the stratification analysis but after that genotype C viruses with BCP double mutations were found to be a particular risk factor for HCC (P = 0.008, OR = 17.19 [95% CI: 2.10 - 140.41]), but those with the wild-type BCP were not. In the interaction analysis, genotype C and BCP double mutations were found to have a synergistic effect on HCC development (P < 0.0001, OR = 52.56 [95% CI: 11.49-240.52]).

**Conclusions:**

The effect of HBV genotype C on the development of HCC differs between wild-type viruses and those with BCP double mutations, suggesting that not all individuals infected with genotype C HBV are at increased risk of HCC.

## 1. Background

HBV infection remains a major public health problem, causing significant morbidity and mortality worldwide (1). Around two billion people, one third of the world’s population, have serological evidence of past or present infection with HBV and, currently, around 350 million people are persistently infected (2). Chronic hepatitis B can lead to chronic active hepatitis, liver cirrhosis and HCC (3).

One of the features of HBV is sequence heterogeneity, because its polymerase lacks proofreading activity (4), consequently eight well-established genotypes (A to H) are recognized, based on >8% intergroup divergence of the complete genome (5-7). The HBV genotypes have distinct geographical distributions (8), and have been reported to have clinical relevance (9). For example, patients with genotypes C and D have a higher risk of disease progression and a poorer clinical outcome compared to the patients with genotypes A and B. Patients with genotypes A and B have a better response to IFN-based therapy than those with genotypes C and D (10). However, the association of genotypes B and C with HCC development remains controversial; some studies have shown that genotype C HBV infection is a risk factor for HCC (11, 12), while others did not obtain the same results (13, 14). The reason to these inconsistencies between the results, especially those derived from the same region (11, 15), are unclear.

Some mutations in HBV genome such as G1896A, C1653T, T1753V, the BCP double mutations (T^1762^A^1764^), and pre-S region deletions have been reported to be associated with HCC development (16, 17). The most convincing association has been detected between HBV and the BCP double mutations which have been confirmed by several prospective studies (18-20). The BCP double mutations occur significantly more frequently in genotype C than genotype B (21, 22). Both genotypes C and BCP double mutations are highly prevalent in HCC (22, 23), and the risk of HCC for patients with either genotype B or C is high if they have BCP mutations (22). Clearly, BCP double mutations are very strong confounding factors of genotypes B and C in HCC development. Therefore, without excluding the confounding effect of BCP double mutations, it is difficult to determine the particular association of genotype C and HCC development.

## 2. Objectives

The aim of this study was to determine whether the effect of HBV genotype C on HCC development differs between those with the BCP double mutations and the wild-type.

## 3. Materials and Methods

### 3.1. Study Subjects and Sample Design

Serum samples were collected from the first affiliated Hospital of Guangxi University of Chinese Medicine and Cancer Institute of Guangxi, including patients with hepatocellular carcinoma (HCC, 107 cases), liver cirrhosis (LC, 89 cases), chronic hepatitis (CH, 96 cases) and HBsAg positive patients attending for reasons not associated with liver disease (asymptomatic carriers([ASC], treated as controls, 110 cases). Diagnostic criteria for ASC were HBsAg positivity for > 6 months and no detectable subjective symptoms, along with persistently (at least 6 months) and normal levels of serum alanine aminotransferase (ALT). CH cases were HBsAg positive for > 6 months, with persistent or intermittent increases of ALT levels, or histological change of the necroinflammatory score (Knodell) ≥ 4. Cirrhosis was diagnosed by histologic analysis of liver biopsy specimens or by ultrasonography findings (i.e. coarse liver architecture, nodular liver surface, and blunt liver edges) and evidences of hypersplenism (i.e. splenomegaly on ultrasonography, and a platelet count of < 100 000/mm^3^). HCC was diagnosed according to the criteria described previously (19). The study protocol conformed to the ethical guidelines of the 1975 Declaration of Helsinki, and was approved by the Guangxi Institutional Review Board. Written informed consents were obtained from the study participants.

### 3.2. Serological Testing

Serological testing was performed in hospitals. Sera were tested for HBV serological markers using enzyme immunoassays (Beijing Wantai Biological Pharmacy Enterprise, Beijing, China). Alanine aminotransferase (ALT) concentrations were determined using a kinetic method (Zhejiang Elikan Biological Technology company, Limited, Wenzhou, Zhejiang, China).

### 3.3. Nested PCR for HBV DNA and Nucleotide Sequencing

DNA was extracted from 85µL serum samples by Pronase digestion followed by phenol/chloroform extraction. The first round amplification protocol and primers P1 and P2 were as described previously (24). The second round PCR was performed on 5 µL of the first round products in a 50 µL reaction using primers LSBI1 (nt 2809-2829, 5'- TTGTGGGTCA CCATATTCTT-3') and HCO2 (nt 761-776, 5'-GCGAAGCTTGCTGTACAGACTTGG- 3') to amplify the preS/S region and CPRF1 (nt 1678–1695, 5' -CAATGTCAACGACCGACC- 3'), and MDN5R (nt 1794–1774, 5' -ATTTATGCCTACAGCCTCCT- 3') to amplify the basal core promoter (BCP). The protocol for the preS/S region is 5 min hot start followed by 30 cycles of 94 ^o^C for 30s, 50 oC for 30s and 72°C for 90s, and for the BCP it included 5 min hot start followed by 30 cycles of 94 ^o^C for 30s, 55 oC for 30 s, and 72oC for 30s. HBV DNA positive products were sent to The Sangon Biotech (Shanghai, China) for sequencing, using a BigDye Terminator V3.1 Cycle Sequencing kit (Applied Biosystems, Foster City, USA) with sequencing primer LSBI1 for the preS/S sequence and CPRF1for BCP sequence.

### 3.4. Measurement of Viral Loads

HBV DNA was extracted with phenol/chloroform extraction and sent to Shanghai ZJ Bio-Tech Co., Ltd. (Shanghai, China) for viral load measurement. The DNA was amplified and quantified in an ABI Prism 7500 sequence detection system (Applied Biosystems, Foster City, CA, California, USA) using HBV primers and a dual labeled TaqMan probe, as described previously (25).

### 3.5. HBV Genotyping

HBV genotypes were determined using the sequences of pre-S/S genes and the STAR program [http://www.vgb.ucl.ac.uk/starn.shtml] (26) and the NCBI Genotyping Tool (http://www.ncbi.nlm.nih.gov/projects/genotyping/formpage.cgi).

### 3.6. Statistical Analysis

The statistical analyses were performed using Pearson’s x^2^ tests. A 95% confidence interval (95% CI) was estimated for the prevalence of viral mutations, and genotypes. Univariate and multivariate logistic regression analyses were performed using the Statistical Package for the Social Sciences (SPSS version 17.0). Variables with P < 0.1 on univariate analysis were analyzed by stepwise multivariate analysis for independent risk factors associated with the development of HCC. All P values were two-tailed and P < 0.05 was considered to be statistically significant.

## 4. Results

### 4.1. Baseline Characteristics

A total number of 402 HBsAg positive cases were recruited for this study. BCP and pre-S sequences were obtained from HBV DNA positive individuals including 97 HCC patients (86 males, 11 females), 72 LC patients (56 males, 16 females), 80 CH patients (59 males, 21 females), and 79 controls (36 males, 43 females). The average age was 44.52 ± 14.1 (mean ± SD). Those from which BCP and pre-S sequences could not be obtained were excluded from the study. The average ages of the HCC, LC, CH and control groups were 48.8 ± 10.8, 38.3 ± 13.0, 38.3 ± 13.0, and 41.1 ± 16.8, respectively. Genotypes B, C and I were found with a prevalence of 31.1% (102/328), 62.8% (206/328), 6.1% (20/328, respectively (Table 1). 

**Table 1.** Genotypes and Liver Disease

Liver Diseases	No.	Genotype B		Genotype C		Genotype I	
		No.	Rate (%)	No.	Rate (%)	No.	Rate (%)
**ASC ** ^**a**^	79	39	49.4 (38.4 - 60.4)	38	48.1 (37.1 - 59.1)	2	2.5 (-0.9 - 5.9)
**CH ** ^**a**^	80	30	37.5 (26.9 - 48.1)	41	51.3 (40.3 - 62.3)	9	11.3 (4.4 - 18.2)
**LC ** ^**a**^	72	19	26.4 (16.2 - 36.6)	48	66.7 (55.8 - 77.6)	5	6.9 (1.1 - 12.8)
**HCC ** ^**a**^	97	14	14.4 (7.4 - 21.4)	79	81.4 (73.7 - 89.1)	4	4.1 (0.2 - 8.1)
**Total**	328	102	31.1 (26.1 - 36.1)	206	62.8 (57.6 - 68.0)	20	6.1 (3.5 - 8.7)

^a^ Abbreviations: ASC, asymptomatic carriers; CH, chronic hepatitis; HCC, hepatocellular carcinoma; LC, liver cirrhosis.

### 4.2. Genotypes and Viral Mutations

The total prevalence of BCP double mutations and pre-S deletions were 60.4% (198/328, 95% CI, 55.1% - 65.7%) and 18.6% (61/328, 95% CI, 14.4% - 22.8%), respectively. The prevalence of BCP double mutations in genotypes B, C and I were 39.2% (40/102, 95% CI, 29.7% - 48.7%), 70.4% (145/206, 95% CI, 64.2% - 76.6%) and 65.0% (13/20, 95% CI, 44.1% - 85.9%), respectively. The prevalence of BCP double mutations in genotypes C and I were significantly higher than that of the genotype B (C vs. B: X^2^ = 27.637, P < 0.001; I vs. B: X^2^ = 4.525, P < 0.05). However, there was no significant difference between genotypes C and I regarding the prevalence of BCP double mutations. The prevalence of pre-S deletions in genotypes B, C and I were 20.6% (21/102, 95% CI, 12.8% - 28.4%), 17.5% (36/206, 95% CI, 12.3% - 22.7%), and 20.0% (4/20, 95% CI, 2.5% - 37.5%), respectively. There was no significant difference in the prevalence of pre-S deletions among genotypes B, C and I.

### 4.3. Genotypes and Viral Loads

High viral load (≥ 10^4^ copies/mL) is a risk factor for HCC (14). The total percentage of high viral loads was 67.1% (220/328). The percentages of high viral loads in genotypes B, C and I were 52.9% (54/102), 72.8% (150/206) and 80.0% (16/20), respectively. The occurrence in genotypes C and I was significantly higher than that of the genotype B (C vs. B: X^2^ = 12.049; I vs. B: X^2^ = 16.564, both P < 0.01), but there was no significant difference in the percentage of high viral loads between genotypes C and I (X^2^ = 0.483, P > 0.05), suggesting that genotypes C and I were associated with high viral loads.

### 4.4. Genotypes and Liver Diseases

The prevalence of genotype B in ASC controls, CH, LC and HCC was 49.4%, 37.5%, 26.4%, and 14.4%, respectively, the prevalence of genotype C in ASC controls, CH, LC and HCC was 48.1%, 51.3%, 66.7%, and 81.4%, respectively, and the prevalence of genotype I in ASC controls, CH, LC and HCC was 2.5%, 11.3%, 6.9%, and 4.1%, respectively (Table 1). Clearly, the prevalence of genotype B decreased with the severity of liver disease, while it increased with the severity of liver disease for genotype C. No trend was found for the less common genotype I.

Multivariate logistic regression analysis was performed to determine whether there is an association between genotype and liver disease. Factors included in the analysis were sex, age, genotypes, viral load, BCP double mutations, pre-S deletion mutations, HBeAg, anti-HBe, and ALT concentration. The results showed that male sex, BCP double mutations, pre-S deletions, HBeAg and abnormal ALT are risk factors of chronic hepatitis and male sex, old ages, BCP double mutations, pre-S deletions, anti-HBe are risk factors of liver cirrhosis. Male gender, old age, BCP double mutations and pre-S deletions are risk factors of HCC (Table 2). Clearly, BCP double mutations are risk factors of advanced liver disease, including CH, LC, and HCC. The association between genotypes, including B, C, and I and specific liver diseases, CH, LC, and HCC was not significant, suggesting that genotypes are not particular risk factors for severe liver disease.

**Table 2.** Complete Analysis of HCC Associated Factors ^a^

Variables	P value	Hazard Ratio	95% CI for Hazard Ratio	
			Lower	Upper
**Univariate Analysis**				
**Sex**				
Female ^b^	-	-	-	-
Male	0.00	9.34	4.33	20.13
**Ages, y**				
< 30 ^b^	0.00	-	-	-
30 - 40	0.001	13.62	2.87	64.7
40 - 50	0.00	39.00	7.52	202.23
50 - 60	0.00	35.5	7.19	174.83
≥ 60	0.00	19.00	3.83	94.29
**Genotypes B ^b^**	0.00	-	-	-
C	0.00	5.79	2.81	11.93
I	0.06	5.57	0.92	33.84
**DNA**				
< 10^4^ ^b^	-	-	-	-
≥ 10^4^	0.04	1.96	1.04	3.67
**BCP Wild-type ^b^**				
Mutations	0.00	41.72	17.19	101.28
**PreS deletions**				
Wild-type ^b^	-	-	-	-
Mutations	0.00	14.10	3.23	61.56
**HBeAg**				
Negative ^b^	-	-	-	-
Positive	0.79	1.10	0.53	2.34
**Anti-HBe**				
Negative ^b^	-	-	-	-
Positive	0.07	1.78	0.96	3.30
**Abnormal ALT**				
Negative ^b^	-	-	-	-
Positive	0.03	2.38	1.10	5.18
**Multivariate Analysis**				
**Sex**	0.00	26.85	6.19	116.46
**Ages, y**				
30 ^b^	0.01	1	-	-
30 - 40	0.00	29.62	3.08	284.64
40 - 50	0.00	56.84	5.65	571.65
50 - 60	0.00	53.14	5.08	555.92
≥ 60	0.01	18.06	1.87	174.80
**BCP mutations**	0.00	66.35	16.32	273.14
**PreS deletions**	0.06	5.52	0.97	31.50

^a^ HBsAg asymptomatic carriers are the controls.

^b^ The variable used for comparison.

### 4.5. The Association Between Genotypes and HCC With Further Stratification Analysis

The above results showed that BCP double mutations are very strong risk factors of HCC and both genotype C and BCP double mutations are highly prevalent in patients with HCC. Some studies have shown that genotype C is associated with HCC development (11, 12) and in the present study, the association between genotype C and HCC was detected to be significant in univariate analysis, but not in multivariate analysis (Table 2). A further stratification analysis was performed according to BCP status and genotypes to exclude the confounding effect of BCP double mutations. According to BCP status, the stratification analysis results showed that genotype C is a risk factor for the HCC development in individuals with BCP double mutations (P = 0.008, OR = 17.19 [95% CI: 2.10 - 140.41]), but not in those with the wild-type BCP (Table 3), while the results from genotype stratification analysis showed that BCP double mutations are risk factors of HCC in both individuals with genotype C and those with genotypes B and I (Table 4). Clearly, BCP double mutations are risk factors of HCC, but the effect of genotype C on HCC development differs between wild-type viruses and those with BCP double mutations, suggesting that not all of individuals infected with genotype C HBV are at increased risk of HCC. 

A further analysis was performed to determine interactions between genotype C and BCP double mutations. The results showed that the risk of genotype C and double mutations combination is strongest for HCC (P < 0.0001, OR = 52.56 [95% CI: 11.49 - 240.52], Table 5), suggesting that genotype C and BCP double mutations have a synergistic effect on HCC development.

**Table 3.** Stratification Analysis for HCC Associated Factors According to the Status of BCP ^a,^ ^b^

Variables	Patients With BCP Double Mutations		Patients With BCP Wild-Type	
	P value	Hazard Ratio (95% CI)	P value	Hazard Ratio (95% CI)
**Univariate Analysis**				
**Sex**				
Female ^b^	-	-	-	-
Male	0.00	11.1 (3.26 - 37.8)	0.99	5E+008 (0.00-)
**Ages, y**				
< 30 ^b^	0.045	-	0.82	-
30 - 40	0.02	25.5 (1.72 - 377.93)	0.99	3E+008 (0.00-)
40 - 50	0.03	10.5 (1.21 - 91.03)	0.99	8E+008 (0.00-)
50 - 60	0.01	20.25 (2.04 - 200.85)	0.99	2E+008 (0.00-)
≥ 60	0.16	4.25 (0.57 - 31.94)	0.99	5E+008 (0.00-)
**Genotypes B ^b^**	-	-	-	-
C	0.00	5.56 (1.60 - 19.26)	0.90	0.91 (0.22 - 3.72)
DNA ^b^				
< 10^4^	-	-	-	-
≥ 10^4^	0.78	1.18 (0.37 - 3.81)	0.18	3.08 (0.59 - 16.00)
**PreS deletions**				
Wild-type ^b^	-	-	-	-
Mutations	0.12	5.38 (0.66 - 42.45)	0.03	17.43 (1.40 - 217.63)
**HBeAg**				
Negative ^b^	-	-	-	-
Positive	0.34	2.80 (0.34 - 23.06)	0.01	6.86 (1.52 - 30.99)
**Anti-HBe**				
Negative ^b^	-	-	-	-
Positive	0.88	0.91 (0.26 - 3.14)	0.69	0.75 (0.18 - 3.06)
**ALT**				
Negative ^b^	-	-	-	-
Positive	0.09	5.93 (0.74 - 47.58)	0.78	0.74 (0.08 - 6.62)
**Multivariate Analysis**				
**Sex**	0.00	24.69 (3.69 - 167.13)	0.99	0.00 (0.00-)
**Ages, y**				
< 30 ^b^	0.01	-	-	-
30 - 40	0.00	820.83 (9.86 - 68309.09)		
40 - 50	0.00	286.29 (10.00 - 8198.95)		
50 - 60	0.00	365.82 (10.91 - 12270.5)		
≥ 60	0.01	48.59 (2.68-880.13)		
**Genotype C**	0.008	17.19 ( 2.10 - 140.41)		
ALT	0.05	13.58 (0.98 - 189.18)	-	-
HBeAg	-	-	0.01	9.60 (1.78 - 51.92)

^a^ HBsAg asymptomatic carriers were the controls.

^b^ The variable used for comparison. Samples with genotype I was not included in the analysis because of the small sample size.

**Table 4.** Stratification Analysis for HCC Associated Factors According to Genotypes ^a^

Variables	Patients With Genotype B and I		Patients With Genotype C	
	P value	Hazard Ratio (95% CI)	P value	Hazard Ratio (95% CI)
**Univariate Analysis**				
**Sex**				
Male	0.00	17.85 (2.17 - 146.9)	0.00	9.49 (3.76 - 23.91)
Female ^b^	-	-	-	-
**Ages, y**				
< 30 ^b^	0.71	-	0.00	-
30 - 40	0.99	5E+008 (0.00-)	0.00	23.14 (4.22 - 126.92)
40 - 50	0.99	1E+009 (0.00-)	0.00	60.00 (8.98 - 400.81)
50 - 60	0.99	1E+009 (0.00-)	0.00	75.00 (11.34 - 495.95)
≥ 60	0.99	1E+009 (0.00-)	0.00	18.00 (3.22 - 100.49)
**BCP Wild-type ^b^**				
Mutations	0.00	15.17 (3.95 - 58.32)	0.00	60.42 (17.25 - 211.58)
**DNA**				
< 10^4^ ^b^	-	-	-	-
≥ 10^4^	0.10	2.73 (0.82 - 9.06)	0.48	1.35 (0.59 - 3.09)
**PreS deletions**				
Wild-type ^b^	-	-	-	-
Mutations	0.99	5E+009 (0.00-)	0.01	6.95 (1.54 - 31.34)
**HBeAg**				
Negative ^b^	-	-	-	-
Positive	0.16	3.90 (0.59 - 25.70)	0.14	0.53 (0.22 - 1.24)
**Anti-HBe**				
Negative ^b^	-	-	-	-
Positive	0.81	1.15 (0.36 - 3.71)	0.02	2.55 (1.15 - 5.65)
**ALT**				
Negative ^b^	-	-	-	-
Positive	0.03	4.58 (1.21 - 17.35)	0.25	1.81 (0.66 - 5.00)
**Multivariate Analysis**				
**Sex**	0.01	43.23 (2.70 - 692.1)	0.00	19.31 (2.75 - 135.82)
**BCP Mutations**	0.00	36.70 (3.84 - 350.7)	0.00	241.62 (12.67 - 4609.3)
**Ages, y**				
< 30 ^b^	-	-	0.01	1
30 - 40			0.00	283.81 (8.07 - 9984.11)
40 - 50			0.00	203.42 (8.84 - 4682.35)
50 - 60			0.00	123.58 (5.80 - 2634.45)
≥ 60			0.03	14.24 (1.33 - 152.58)

^a^ HBsAg asymptomatic carriers were the controls.

^b^ The variable used for comparison.

**Table 5.** Analysis of the Interactions Between Genotype C and Core Promoter Double Mutations on HCC Development ^a^

Variables	P value	Hazard Ratio	95% CI for Hazard Ratio	
			Lower	Upper
**Univariate Analysis**				
**Sex**				
Male	0.00	9.96	4.50	22.05
Female ^b^				
**Ages**				
< 30 ^b^	0.00	-	-	-
30 - 40	0.00	13.00	2.73	61.88
40 - 50	0.00	42.71	8.05	226.59
50 - 60	0.00	33.09	6.69	163.64
≥ 60	0.00	20.58	4.12	102.93
**DNA**				
< 10^4^ ^b^	-	-	-	-
≥ 10^4^	0.04	1.93	1.03	3.65
**Genotype C-BCP**				
Negative ^b^	-	-	-	-
Positive ^c^	0.00	31.48	13.26	74.76
**PreS deletions**				
Wild-type ^b^	-	-	-	-
Mutations	0.00	13.79	3.15	60.40
**HBeAg**				
Negative ^b^	-	-	-	-
Positive	0.75	1.13	0.54	2.40
**Anti-HBe**				
Negative ^b^				
Positive	0.08	1.75	0.93	3.27
**ALT**				
Negative ^b^	-	-	-	-
Positive	0.02	2.60	1.16	5.81
**Multivariate Analysis**				
**Sex**	0.00	31.27	5.80	168.63
**Ages, y**				
30 ^b^	0.00	-	-	-
30 - 40	0.00	37.84	3.68	389.59
40 - 50	0.00	117.75	10.10	1373.03
50 - 60	0.00	94.29	7.90	1125.69
≥ 60	0.00	38.28	3.49	419.50
**Genotype C-BCP**	0.00	52.56	11.49	240.52
**PreS deletions**	0.06	5.21	0.93	29.18

^a^ HBsAg asymptomatic carriers were the controls.

^b^ The variable used for comparison.

^c^ Positive: individuals with both genotype C and core promoter double mutations.

### 4.6. Prevalence of HBV Genotypes in HCC Patients and ASC Controls According to Age

**Figure 1.**
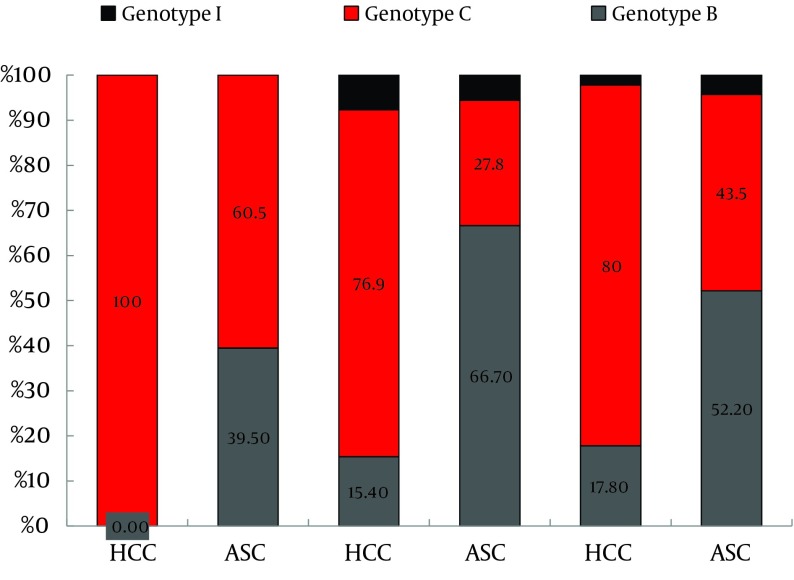
Age-Associated Prevalence of HBV Genotypes in HCC Patients and ASC Controls The prevalence of each genotype in HCC or ASC and among different age groups is shown. There was only one genotype in HCC in the young age group (≤ 35). Genotype I was not found in this age group.

Totally, 16.5% (13/79) of genotype C HBV infected HCC cases were younger than 35 years old. However, none of the genotype B or I infected HCC cases were in this age group. Not only HCC patients aged 35 to 50, but also those older than 50 years had a higher prevalence of genotype C than the ASC controls (X^2^ = 12.551, P < 0.001; X^2^ = 9.276, P < 0.01, respectively). In contrast, not only the HCC patients aged 35 to 50, also those older than 50 years had a significantly lower prevalence of genotype B than the ASC controls (X^2^ = 14.990, P < 0.001; X^2^ = 8.674, P < 0.01, respectively) (Figure 1). 

The incidence of HCC in genotype I (4/6) was higher than genotype B (14/38), although the difference was not significant (X^2^ = 0.873, P > 0.05). In individuals infected with genotype C, the incidence of HCC in the group35-50 years (30/35) was significantly higher than those younger than 35 years (13/36, X^2^ = 18.282, P < 0.001). However, HCC incidence did not differ significantly between the age group of 35-50 and those older than 50 years in genotype C, and it was not different among age groups (< 35, 35 - 50 and > 50) in genotype B either.

## 5. Discussion

The major finding of this study was to confirm if the effect of HBV genotype C on HCC development differs between those with basal core promoter double mutations (T^1762^A^1764^) and the wild-type. Genotype C and BCP double mutations play a synergistic role in HCC development. HCC occurred at a young age (< 35 years old) in some patients with genotype C infection, but no HCC cases infected with genotype B or I were seen in the same age group. The prevalence of BCP double mutations in genotypes C and I was significantly higher than that of genotype B. The proportion of high viral loads (≥ 10^4^ copies/mL) in genotypes C and I was also significantly higher than genotype B. The strength of this study was to perform multivariate logistic regression analysis, not only for the entire data set, but also for the data stratified according to BCP status, genotypes and the interaction between genotype C and BCP double mutations. One of the limitations to this study was the small sample size of genotype I, which limited further stratification analysis of that rare recombinant.

Many published studies have addressed an association between HBV genotype and HCC. Some of these studies showed that HBV genotype C is associated with HCC development, but did not consider the potentially confounding effect of BCP double mutations (12, 27). Other studies showed that genotype C was a significant risk factor of HCC according to univariate analysis, but not in the multivariate analysis (14, 18, 28). A later prospective study from the same institute adjusted for the confounding effect of BCP mutations found that HBV genotype C and BCP double mutations were associated with a high risk of HCC (3). However, the authors did not determine whether the associations of genotype C and HCC differed between those with and without BCP double mutations. The present study was the first one in which multivariate logistic regression analysis was performed for the data stratified according to BCP status. When the analysis was only applied to the entire data set, the association between genotype C and HCC could not be seen, and it was not until after the data stratified analysis was performed according to BCP status, that the association between genotype C and HCC became clear in those with BCP double mutations, but not in those with BCP wild-type.

Individuals infected with HBV genotype C and the BCP double mutation had the highest incidence of HCC (3). The results from the study demonstrated that genotype C and BCP double mutations had a synergistic effect on HCC development. Therefore, it is not difficult to claim that the effect of HBV genotype C on the development of HCC differs between those with core promoter double mutations and the wild-type. Among those infected with HBV with BCP double mutations only, the effect of genotype C on HCC development is strongest, because genotype C and BCP double mutations have a synergistic effect. Therefore, under such circumstances, the risk of genotype C reaches a significant level. However, among those infected with BCP wild-type viruses only, the effect of genotype C is weakest and genotype C does not appear as a risk factor. Among those infected with BCP double mutations or the wild-type, the effect of genotype C is mild. Therefore, sometimes genotype C seems to be a risk factor of HCC, and sometimes not. That is why some researchers have reported that genotype C is a risk factor of HCC, while some did not.

Many studies have suggested that genotype C HBV infection is an independent risk factor for HCC (11, 12), and that it may be used as a marker to identify chronic HBV infected individuals at increased risk of HCC (10). Considering the fact that the effect of HBV genotype C on the development of HCC differs between those with core promoter double mutations and wild-type, this may be the case in those infected HBV with BCP double mutations, but not in those with BCP wild-type. Therefore, our results are important to guide the identification of chronic HBV infected individuals at increased risk of HCC.

Kao et al. reported that genotype B was associated with HCC in patients younger than 35 years, and genotype C was not found in HCC patients in the same age group (27). In contrast, our results showed that 16.5% (13/79) of genotype C HBV infected HCC cases were younger than 35 years, but no genotype B HBV infected HCC cases were in this age group. Therefore, the association of genotype and age in diagnosis of HCC requires further investigations. As others (12) reported, we also found that hepatitis B viral load was higher in patients with genotype C than in ones with genotype B.

Genotype I was recently proposed as a new genotype (29, 30). It has a wide distribution in Asia and has been reported in Vietnam (30, 31), Laos (30) and India (32), as well as south (33), northwest (34) and west (35) of China. However, detailed clinical data of infections with this genotype are lacking. The study, was the first to show that genotype I is not a particular risk for severe liver disease. Genotypes I and C had similar features in viral loads, viral mutations and incidence of HCC, which may be due to sharing same sequences (33). However, this finding requires confirmation, because a small number of genotype I cases were studied in this investigation.

## References

[1] Franco E, Bagnato B, Marino MG, Meleleo C, Serino L, Zaratti L (2012). Hepatitis B: Epidemiology and prevention in developing countries.. World J Hepatol..

[2] Kao JH, Chen DS (2002). Global control of hepatitis B virus infection.. Lancet Infect Dis..

[3] Yang HI, Yeh SH, Chen PJ, Iloeje UH, Jen CL, Su J (2008). Associations between hepatitis B virus genotype and mutants and the risk of hepatocellular carcinoma.. J Natl Cancer Inst..

[4] Steinhauer DA, Holland JJ (1986). Direct method for quantitation of extreme polymerase error frequencies at selected single base sites in viral RNA.. J Virol..

[5] Kramvis A, Restorp K, Norder H, Botha JF, Magnius LO, Kew MC (2005). Full genome analysis of hepatitis B virus genotype E strains from South-Western Africa and Madagascar reveals low genetic variability.. J Med Virol..

[6] Kurbanov F, Tanaka Y, Mizokami M (2010). Geographical and genetic diversity of the human hepatitis B virus.. Hepatol Res..

[7] Okamoto H, Tsuda F, Sakugawa H, Sastrosoewignjo RI, Imai M, Miyakawa Y (1988). Typing hepatitis B virus by homology in nucleotide sequence: comparison of surface antigen subtypes.. J Gen Virol..

[8] Huy TT, Sall AA, Reynes JM, Abe K (2008). Complete genomic sequence and phylogenetic relatedness of hepatitis B virus isolates in Cambodia.. Virus Genes..

[9] Kao JH (2002). Hepatitis B viral genotypes: clinical relevance and molecular characteristics.. J Gastroenterol Hepatol..

[10] Lin CL, Kao JH (2011). The clinical implications of hepatitis B virus genotype: Recent advances.. J Gastroenterol Hepatol..

[11] Chan HL, Hui AY, Wong ML, Tse AM, Hung LC, Wong VW (2004). Genotype C hepatitis B virus infection is associated with an increased risk of hepatocellular carcinoma.. Gut..

[12] Yu MW, Yeh SH, Chen PJ, Liaw YF, Lin CL, Liu CJ (2005). Hepatitis B virus genotype and DNA level and hepatocellular carcinoma: a prospective study in men.. J Natl Cancer Inst..

[13] Sumi H, Yokosuka O, Seki N, Arai M, Imazeki F, Kurihara T (2003). Influence of hepatitis B virus genotypes on the progression of chronic type B liver disease.. Hepatology..

[14] Yuen MF, Tanaka Y, Shinkai N, Poon RT, But DY, Fong DY (2008). Risk for hepatocellular carcinoma with respect to hepatitis B virus genotypes B/C, specific mutations of enhancer II/core promoter/precore regions and HBV DNA levels.. Gut..

[15] Yuen MF, Sablon E, Yuan HJ, Wong DK, Hui CK, Wong BC (2003). Significance of hepatitis B genotype in acute exacerbation, HBeAg seroconversion, cirrhosis-related complications, and hepatocellular carcinoma.. Hepatology..

[16] Liu CJ, Chen BF, Chen PJ, Lai MY, Huang WL, Kao JH (2006). Role of hepatitis B virus precore/core promoter mutations and serum viral load on noncirrhotic hepatocellular carcinoma: a case-control study.. J Infect Dis..

[17] Liu S, Zhang H, Gu C, Yin J, He Y, Xie J (2009). Associations between hepatitis B virus mutations and the risk of hepatocellular carcinoma: a meta-analysis.. J Natl Cancer Inst..

[18] Chu CM, Lin CC, Lin SM, Lin DY, Liaw YF (2012). Viral load, genotypes, and mutants in hepatitis B virus-related hepatocellular carcinoma: special emphasis on patients with early hepatocellular carcinoma.. Dig Dis Sci..

[19] Fang ZL, Sabin CA, Dong BQ, Ge LY, Wei SC, Chen QY (2008). HBV A1762T, G1764A mutations are a valuable biomarker for identifying a subset of male HBsAg carriers at extremely high risk of hepatocellular carcinoma: a prospective study.. Am J Gastroenterol..

[20] Yuen MF, Tanaka Y, Fong DY, Fung J, Wong DK, Yuen JC (2009). Independent risk factors and predictive score for the development of hepatocellular carcinoma in chronic hepatitis B.. J Hepatol..

[21] Du H, Li T, Zhang HY, He ZP, Dong QM, Duan XZ (2007). Correlation of hepatitis B virus (HBV) genotypes and mutations in basal core promoter/precore with clinical features of chronic HBV infection.. Liver Int..

[22] Kao JH, Chen PJ, Lai MY, Chen DS (2003). Basal core promoter mutations of hepatitis B virus increase the risk of hepatocellular carcinoma in hepatitis B carriers.. Gastroenterology..

[23] Yuan J, Zhou B, Tanaka Y, Kurbanov F, Orito E, Gong Z (2007). Hepatitis B virus (HBV) genotypes/subgenotypes in China: mutations in core promoter and precore/core and their clinical implications.. J Clin Virol..

[24] Gunther S, Li BC, Miska S, Kruger DH, Meisel H, Will H (1995). A novel method for efficient amplification of whole hepatitis B virus genomes permits rapid functional analysis and reveals deletion mutants in immunosuppressed patients.. J Virol..

[25] Fang ZL, Sabin CA, Dong BQ, Wei SC, Chen QY, Fang KX (2009). The association of HBV core promoter double mutations (A1762T and G1764A) with viral load differs between HBeAg positive and anti-HBe positive individuals: a longitudinal analysis.. J Hepatol..

[26] Myers R, Clark C, Khan A, Kellam P, Tedder R (2006). Genotyping Hepatitis B virus from whole- and sub-genomic fragments using position-specific scoring matrices in HBV STAR.. J Gen Virol..

[27] Kao JH, Chen PJ, Lai MY, Chen DS (2000). Hepatitis B genotypes correlate with clinical outcomes in patients with chronic hepatitis B.. Gastroenterology..

[28] Yuen MF, Tanaka Y, Mizokami M, Yuen JC, Wong DK, Yuan HJ (2004). Role of hepatitis B virus genotypes Ba and C, core promoter and precore mutations on hepatocellular carcinoma: a case control study.. Carcinogenesis..

[29] Olinger CM, Jutavijittum P, Hubschen JM, Yousukh A, Samountry B, Thammavong T (2008). Possible new hepatitis B virus genotype, southeast Asia.. Emerg Infect Dis..

[30] Tran TT, Trinh TN, Abe K (2008). New complex recombinant genotype of hepatitis B virus identified in Vietnam.. J Virol..

[31] Hannoun C, Norder H, Lindh M (2000). An aberrant genotype revealed in recombinant hepatitis B virus strains from Vietnam.. J Gen Virol..

[32] Arankalle VA, Gandhe SS, Borkakoty BJ, Walimbe AM, Biswas D, Mahanta J (2010). A novel HBV recombinant (genotype I) similar to Vietnam/Laos in a primitive tribe in eastern India.. J Viral Hepat..

[33] Fang ZL, Hue S, Sabin CA, Li GJ, Yang JY, Chen QY (2011). A complex hepatitis B virus (X/C) recombinant is common in Long An county, Guangxi and may have originated in southern China.. J Gen Virol..

[34] Yu H, Yuan Q, Ge SX, Wang HY, Zhang YL, Chen QR (2010). Molecular and phylogenetic analyses suggest an additional hepatitis B virus genotype "I".. PLoS One..

[35] Kang WY, Bi SL, Ding ZR, Tian BJ, Zhao ZX, Li H (2011). [Molecular identification of hepatitis B virus genotype I in Yunnan Province, the People's Republic of China].. Bing Du Xue Bao..

